# 10.D. Workshop: Injury Severity Classification and burden of disability measurement

**DOI:** 10.1093/eurpub/ckac129.631

**Published:** 2022-10-25

**Authors:** Organised by: EUPHA-INJ, EUPHA-EPI, EUPHA-PHMR, EUPHA-ECO, Chair persons: Maria Papadakaki (EUPHA-INJ), João Vasco Santos (EUPHA-ECO)

## Abstract

Globally, injuries cause over 4.3 million deaths per year with incalculable costs for the health, legal, and social systems. Burden of disease analyses are being conducted across the world in order to map the health status of injured population, run comparisons and enable prioritization of preventive and interventional measures. Metrics of injury burden are therefore important indicators of population health that are increasingly being incorporated into national and international health information systems. To prevent injuries and their disabling health consequences, and to effectively reduce injury burden, it is essential to provide policy makers and health system planners with detailed measurements and assessments that allow them to focus resources where they are most needed and areas where more targeted policy attention might be required. However, efforts to fully understand the health outcomes of trauma patients remain inconsistent and insufficient, specifically for certain injury populations and health outcome domains. A wide variety of measures are available to track outcomes after injury in various health domains. Reaching consensus is an important step in benchmarking of outcomes across institutions and injury types to improve quality and advance the field of injury care. Modern, validated measures that are feasible and usable in both research and clinical contexts are needed to facilitate the improvement of quality and comparability of research. The workshop aims to discuss trends and variation of injury burden, which is critical to health system planning. The high human and societal costs as well as inequalities of injury mortality and burden will be highlighted based on comparisons with long-term trends. The workshop will discuss the latest developments in injury severity classification and disability measurement. The most efficient measures to calculate the valid burden of injuries will be presented and comparative measurements across EU countries will be promoted. Limitations of injury classifications and factors introducing uncertainty and potential bias in estimation of disability, will be identified. The workshop will contribute to the identification of evidence-informed tools and measurements of injury burden.

**Key messages:**

• Some injury outcomes are responsible for huge individual and societal burden but are still difficult to measure.

• Multiple measures are developed to assess severity and outcomes: e.g. AIS/ ISS/New ISS/ ICD-10. Efficient prevention and quality care requires validated and easy to use measures.

